# Correction: Synthesis and characterization of AFe_2_O_4_ (A: Ni, Co, Mg)–silica nanocomposites and their application for the removal of dibenzothiophene (DBT) by an adsorption process: kinetics, isotherms and experimental design

**DOI:** 10.1039/d2ra90075k

**Published:** 2022-07-21

**Authors:** Fahimeh Vafaee, Samira Mandizadeh, Omid Amiri, Mansour Jahangiri, Masoud Salavati-Niasari

**Affiliations:** Faculty of Chemical, Petroleum and Gas Eng., Semnan University P. O. Box 35196-45399 Semnan Islamic Republic of Iran mjahangiri@semnan.ac.ir +98 31 55913201 +98 31 55912383; Institute of Nano Science and Nano Technology, University of Kashan P. O. Box 87317-51167 Kashan I. R. Iran salavati@kashanu.ac.ir; Faculty of Chemistry, Razi University Kermanshah 6714414971 Iran; Department of Chemistry, College of Science, University of Raparin Rania Kurdistan Region Iraq

## Abstract

Correction for ‘Synthesis and characterization of AFe_2_O_4_ (A: Ni, Co, Mg)–silica nanocomposites and their application for the removal of dibenzothiophene (DBT) by an adsorption process: kinetics, isotherms and experimental design’ by Fahimeh Vafaee *et al.*, *RSC Adv.*, 2021, **11**, 22661–22676, https://doi.org/10.1039/D1RA02780H.

The authors regret an error in Fig. 4 where a section of the XRD for 4(a) and (b) is identical.

The authors have repeated the experiment and provided new data for Fig. 4. An independent expert has viewed the new data and has concluded that it is consistent with the discussions and conclusions presented. The correct Fig. 4 is shown below:

**Fig. 4:**
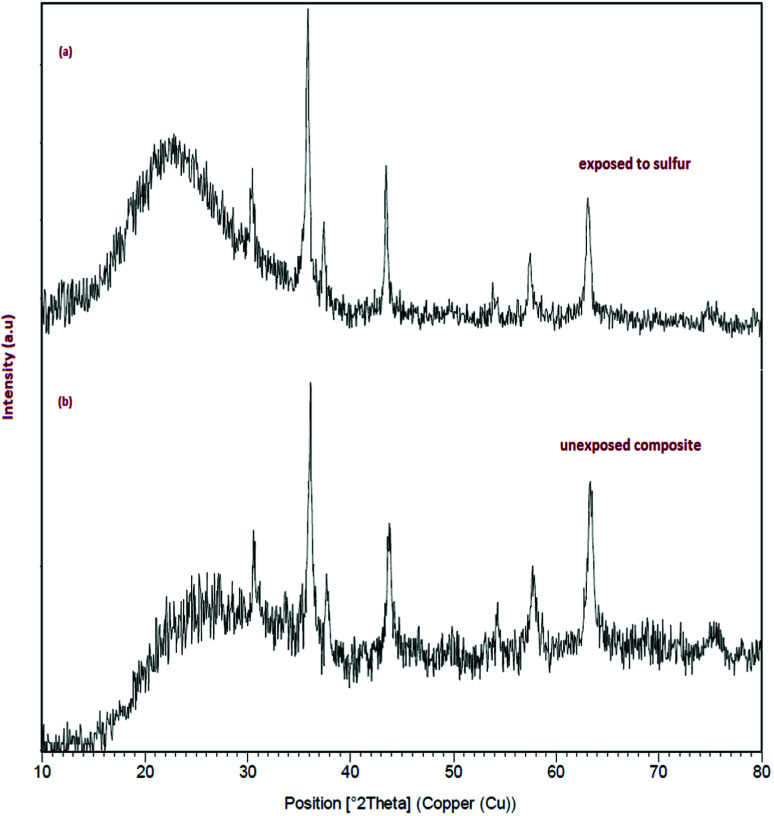
(a) The XRD pattern of sample 3 after adsorption of DBT. (b) The XRD pattern of sample 3 before adsorption of DBT.

The Royal Society of Chemistry apologises for these errors and any consequent inconvenience to authors and readers.

## Supplementary Material

